# Identification of potential vaccine targets for COVID‐19 by combining single‐cell and bulk TCR sequencing

**DOI:** 10.1002/ctm2.430

**Published:** 2021-05-21

**Authors:** Pingping Wang, Zhaochun Xu, Wenyang Zhou, Xiyun Jin, Chang Xu, Meng Luo, Kexin Ma, Huimin Cao, Yan Huang, Xiaoyu Lin, Fenglan Pang, Yiqun Li, Qinghua Jiang

**Affiliations:** ^1^ Center for Bioinformatics School of Life Science and Technology Harbin Institute of Technology Harbin China; ^2^ Key Laboratory of Biological Big Data, Harbin Institute of Technology Ministry of Education Harbin China

Dear Editor,

COVID‐19 is a highly infectious novel pneumonia that has become the largest health crisis in modern history. T cells participate in recognizing and clearing viral infections and also helping B cells to produce antibodies. This pivotal role of T cells in immunity makes them ideal targets for studying the immune response in COVID‐19. Here, we use scRNA‐seq, scTCR‐seq, deep TCR‐seq, and HLA‐genotyping to decode the matching T cell phenotype and antigenic epitopes of 16 early‐recovery COVID‐19 patients in the context of human HLA haplotypes.

We collected fresh blood samples from 16 early‐recovery patients with COVID‐19 (Table S1). PBMCs were isolated for subsequent data generation: (1) single‐cell transcriptome sequencing, (2) single‐cell TCR sequencing, (3) deep TCR repertoire sequencing, and (4) HLA genotyping (Figure 1A and Table S1). Overall, we totally obtained single cell gene expression data from 26 223 T cells, single cell paired αβTCRs from 27 467 T cells and hypervariable regions of immune receptors from 4.9 million TCR clones (Table S2). In addition, high‐resolution HLA typing results were performed by sequencing 5 HLA genes, including HLA‐A, B, C, DRB1, and DQB1 (Table S3). In order to reveal the T cell response changes caused by COVID‐19, 8 healthy controls (healthy cohort1) with scRNA‐seq profiled by 10x Genomics and 31 healthy controls (healthy cohort2) with deep TCR‐seq were included in this study (Figure S1).

**FIGURE 1:**
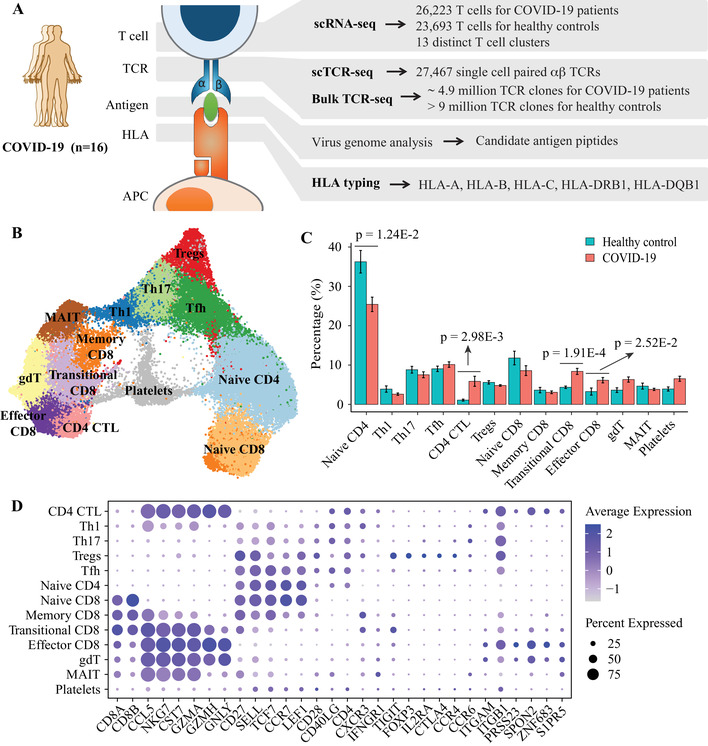
Single cell transcriptome profiling of T cells of patients with COVID‐19 and controls. (A) An overview of experimental design. PBMCs from 16 patients with COVID‐19 were divided to perform scRNA‐seq, scTCR‐seq, deep TCR‐seq and HLA genotyping. (B) UMAP plot of T cells from patients with COVID‐19 and controls. Clustering was based on unsupervised k‐means using the normalized gene expression values after batch effect removal. (C) Bar plots showing the distribution of T cell types in patients with COVID‐19 and healthy controls. (D) Dot plot shows the average log‐normalized gene expression of marker genes for cell types discussed in the main text. The size of the dot represents the percentage of cells that express the gene in each cluster, and the color represents the average level of expression after scaling

Using unsupervised clustering, we identified 13 distinct T cell types (Figure 1B), including naïve CD4+ T cells (Naïve CD4), Th1 cells (Th1), Th17 cells (Th17), Tfh cells (Tfh), cytotoxic CD4+ T cells (CD4 CTL), regulatory T cells (Tregs), naïve CD8+ T cells (Naïve CD8), memory CD8+ T cells (Memory CD8), transitional CD8+ T cells (transitional CD8), terminal effector CD8+ T cells (Effector CD8), gamma‐delta T cells (gdT), MAIT cells (MAIT), and platelet‐like cells (Platelets). Cells were annotated by SingleR.^1^ Classical marker genes were used to distinguish different cell types (Figure 1D, Figure S2).

Of all the T cell types identified in this study, the proportion of Transitional CD8, Effector CD8 and CD4 CTL in COVID‐19 patients were significantly higher than those in healthy controls (*P* = 1.91 × 10^−4^, 2.52 × 10^−2^, and 2.98 × 10^−3^, respectively), while the proportion of Naïve CD4 was significantly lower (*P* = 1.24 × 10^−2^) (Figure 1C). Single cell repertoire analysis demonstrates that larger clonotypes exhibited a non‐uniform distribution of cell types with an enrichment for cytotoxic T cells, such as transitional CD8, effector CD8 and CD4 CTL (Figure 2). Interestingly, effector CD8 and CD4 CTL in our study also jointly expressed resident memory marker ZNF683^2^ and tissue exit marker S1RP5^3^ (Figure 1D). According to Ref. (4), these cells might be cytotoxic T cells recently egressed from tissues (such as lung tissue) and reentered circulation, an observation that waits for further experimental validation.

**FIGURE 2:**
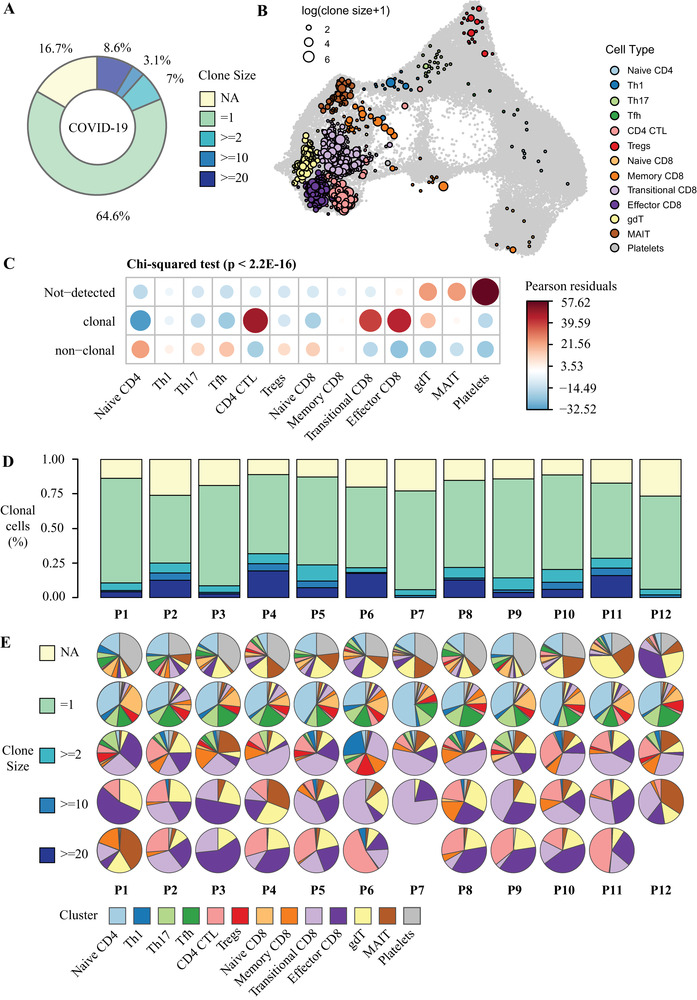
Clonally expanded T cells in COVID‐19 patients. (A) Clonal distribution of T cell receptors in COVID‐19 patients. (B) UMAP plot shows the distribution of clonally expanded T cells. (C) Residual plot for the Pearson's chi‐squared test of clone expansion from cell types, using the corrplot package in R. Red circles indicate an overrepresentation, and blue circles indicate an underrepresentation. A Pearson's chi‐squared test shows this difference is statistically significant (*χ* ^2^ = 17605, df = 24, simulated *P* < 2.2E‐16). (D) Bar plot shows the distribution of clonotypes by size (NA = 1, ≥ 2, ≥ 10 ,and ≥ 20 cells, NA represents cells with no αβTCR sequence detected). (E) Pie charts show the cell type composition of clonotypes from each sample stratified by clone size

To investigate whether the TCR repertoires of the recovered patients with COVID‐19 differentiate from healthy individuals, we compared the deep TCR‐seq data between patients with COVID‐19 and controls. At the repertoire level, T cell diversity in COVID‐19 patients was significantly lower than that in controls (*P* < 0.0001, Figure 3A), consistent with the clonal expansion upon antigen exposure. Clustering of TCRs with similar CDR3s is an effective approach to identify antigen‐specific T cells,^5^, ^6^, ^7^ as TCRs sharing similar motifs from distinct individuals may also share antigen‐specificity. Through TCR clustering, we detected 29 409 TCR groups (Table S4). Interestingly, COVID‐19 patients shared more TCR groups than healthy controls (Figure 3B). To obtain patient‐specific TCRs, we searched for CDR3 groups significantly enriched in the COVID‐19 cases, and identified a total of 916 groups (FDR < 0.05, Table S5), which were referred as ‘COVID‐19 TCR groups’ in the downstream analysis. These groups of T cells are enriched for activated T cells, specifically, the transitional CD8, effector CD8 and CD4 CTL subtypes (Figure 3C‐D).

**FIGURE 3:**
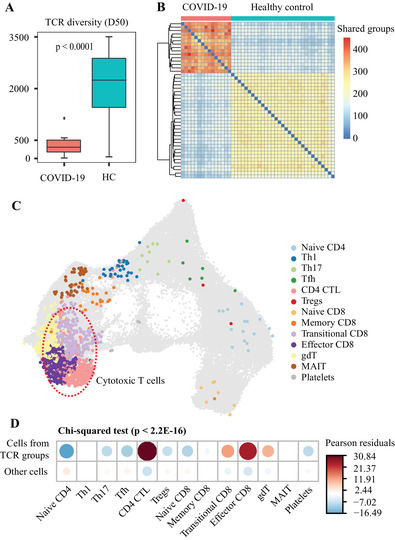
Antigen‐specific T cells in COVID‐19 patients. (A) Comparison of D50 TCR diversity between patients with COVID‐19 and healthy controls. Low D50 value indicates low diversity and high clonal expansion. (B) Heatmap plot for clustering results of shared TCR groups among all samples. Each entry of the pairwise sharing matrix documents the number of shared TCR groups between two individuals. Unsupervised hierarchical clustering was applied to organize the columns and rows of the matrix. (C) The distribution of T cells of 916 COVID‐19 TCR groups overlaid on top of all the single cell data in the UMAP plot of T cells. (D) Residual plot for the Pearson's chi‐squared test of COVID‐19 specific TCR groups from cell types, using the corrplot package in R. Red circles indicate an overrepresentation, and blue circles indicate an underrepresentation. A Pearson's chi‐squared test shows this difference is statistically significant (*χ* ^2^ = 2658.5, df = 12, simulated *P* < 2.2E‐16)

The identification of COVID‐19 TCR groups also allowed us to uncover the candidate antigenic epitopes from the virus genome. In total, 866 9‐mer peptides from 11 SARV‐CoV‐2 proteins were computationally predicted to bind patient HLA alleles profiled in our study (Table S6). We examined the peptides and CDR3 groups found in multiple individuals, and identified 1602 cooccurring TCR‐antigen pairs that were significantly shared by the same patients, covering 31 CDR3 groups and 114 peptides (FDR < 0.05, Table S7). Of these, we identified two pairs, each with a single TCR group and a single antigen (FDR < 0.001, Figure 4A). Another pair consists of one CDR3 group mapping to multiple epitopes (Figure 4A), which demonstrated similar motifs in the TCR contact regions.^8^ We next located all the 114 peptides in the virus genome, and found more than 91% were distributed in proteins ORF1ab, S, N, and ORF3a (Figure 4B), where ORF3a showed significant epitope enrichment (*P* = 0.0056, Binomial test). In summary, our analysis revealed a number of candidate peptides as promising targets for COVID‐19 vaccine development.

**FIGURE 4:**
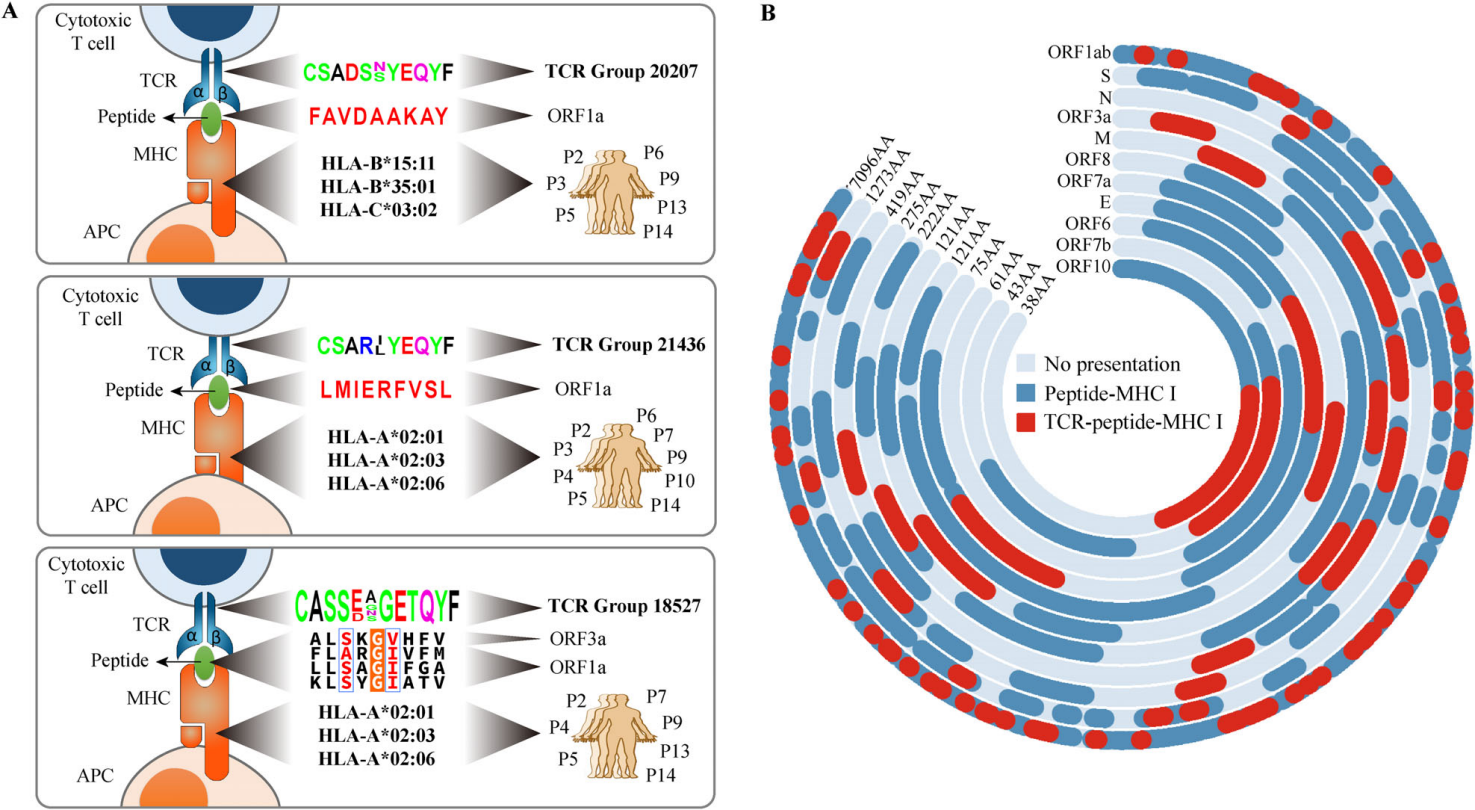
Identification of clonally expanded TCR groups and potential virus epitopes. (A) Diagram showing perfectly matched TCR groups and peptides presented by MHC I alleles of COVID‐19 patients (FDR < 0.001). (B) The distribution of MHC I presentation regions in each protein from the SARS‐CoV‐2 genome. Peptides computationally predicted to bind MHC‐I with high affinity are colored as dark blue. Those peptides presented by MHC‐I with high affinity, significantly cooccurring with a COVID‐19 TCR group are colored as red

This study provides an effective solution for identifying potential antigenic peptides based on large‐scale TCR repertoire and HLA typing. The combined use of single‐cell and deep TCR sequencing provided us with single‐cell resolution, and also enabled us to obtain millions of immune receptors.^9^ However, highly abundant T cell clones may not be disease‐specific.^10^ With this strategy, we grouped similar TCRs to search for evidence of convergent selection in patients. Mapping these receptors to single cell data identified novel T cell phenotypes specific to recovery COVID‐19 patients. In addition, with HLA genotyping, we were able to provide individualized TCR epitopes, which allowed us to investigate their associations with recurrent TCR groups across different individuals. This method led to statistically confident antigen targets and provided guidance for efficient mRNA vaccine design. We hope that our findings and immune receptor datasets will inform the development of next‐generation vaccines that can better activate natural T cell immunity for COVID‐19.

## COMPETING INTERESTS

The authors declare no conflict of interest.

## DATA AVAILABILITY STATEMENT

Single cell transcriptome sequencing data were deposited on Zenodo (https://doi.org/10.5281/zenodo.3747336). Custom scripts in this study are available upon request to the corresponding author.

## AUTHOR CONTRIBUTIONS

Qinghua Jiang conceived the project. Pingping Wang, Zhaochun Xu and Wenyang Zhou performed the bioinformatic analyses. Qinghua Jiang, Pingping Wang and Zhaochun Xu wrote the manuscript. Xiyun Jin, Chang Xu, Meng Luo, Kexin Ma, Huimin Cao discussed the results and commented on the manuscript. Yan Huang, Xiaoyu Lin, Fenglan Pang and Yiqun Li revised the manuscript.

Pingping Wang, Zhaochun Xu, Wenyang Zhou contributed equally as first authors.

## ETHICS APPROVAL AND CONSENT TO PARTICIPATE

Ethical approval for this study was obtained from the Ethics Committee in the Harbin sixth Hospital (Approval number: 2020NO.12). Written informed consent was obtained from all participants.

## Supporting information

Supporting information

Supporting information

Supporting information

Supporting information

Supporting information

Supporting information

Supporting information

Supporting information

Supporting information

Supporting information

Supporting information

Supporting information
