# Supplementary material for: Identification of potential vaccine targets for COVID‐19 by combining single‐cell and bulk TCR sequencing
Source: Clin Transl Med. 2021 May 21;11(5):e430. doi: 10.1002/ctm2.430 (PMC8140189; doi:10.1002/ctm2.430)
Supplement: Supplementary file 1 — Supporting information [file CTM2-11-e430-s012.pdf]

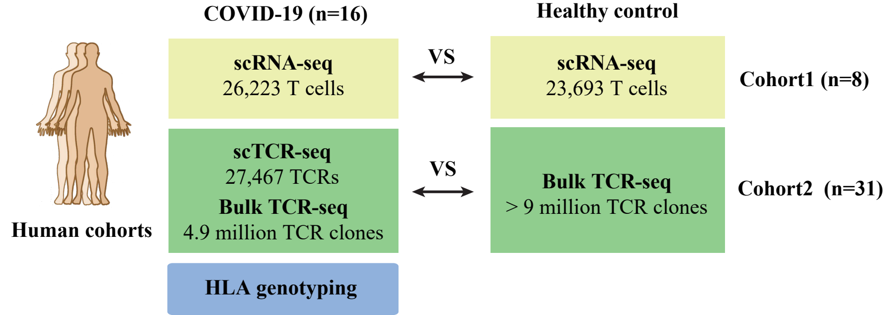
COVID-19 (n=16)
Healthy control
scRNA-seq
26,223 T cells
VS
scRNA-seq
23,693 T cells
Cohort1 (n=8)
scTCR-seq
27,467 TCRs
Bulk TCR-seq
4.9 million TCR clones
VS
Bulk TCR-seq
> 9 million TCR clones
Cohort2 (n=31)
Human cohorts
HLA genotyping
