# Supplementary figures and images for: Identification of potential vaccine targets for COVID‐19 by combining single‐cell and bulk TCR sequencing
Source: Clin Transl Med. 2021 May 21;11(5):e430. doi: 10.1002/ctm2.430 (PMC8140189; doi:10.1002/ctm2.430)

CD4

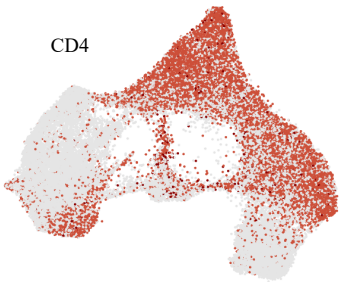

CD8A

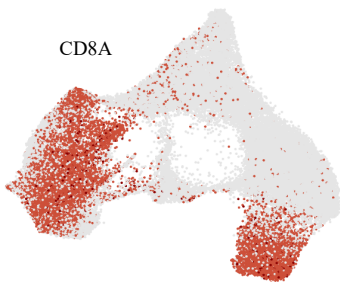

CCR7

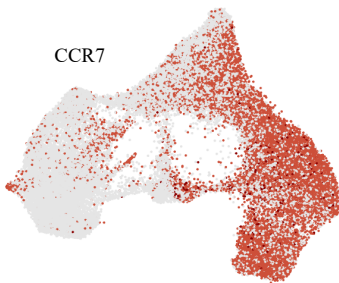

FOXP3

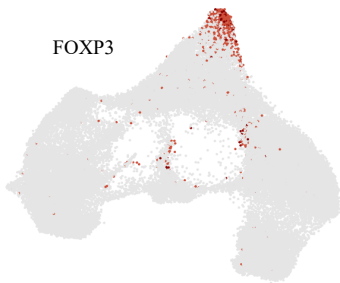

GZMA

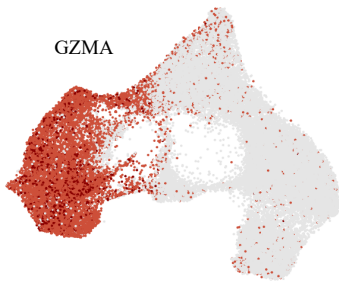

GZMK

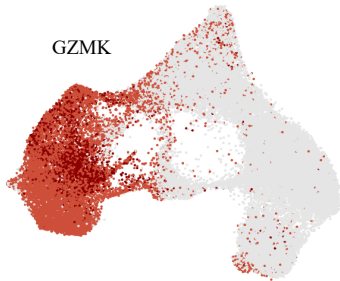

Supplement: Supplementary file 2 — Supporting information [file CTM2-11-e430-s008.pdf]

Number of missense variants

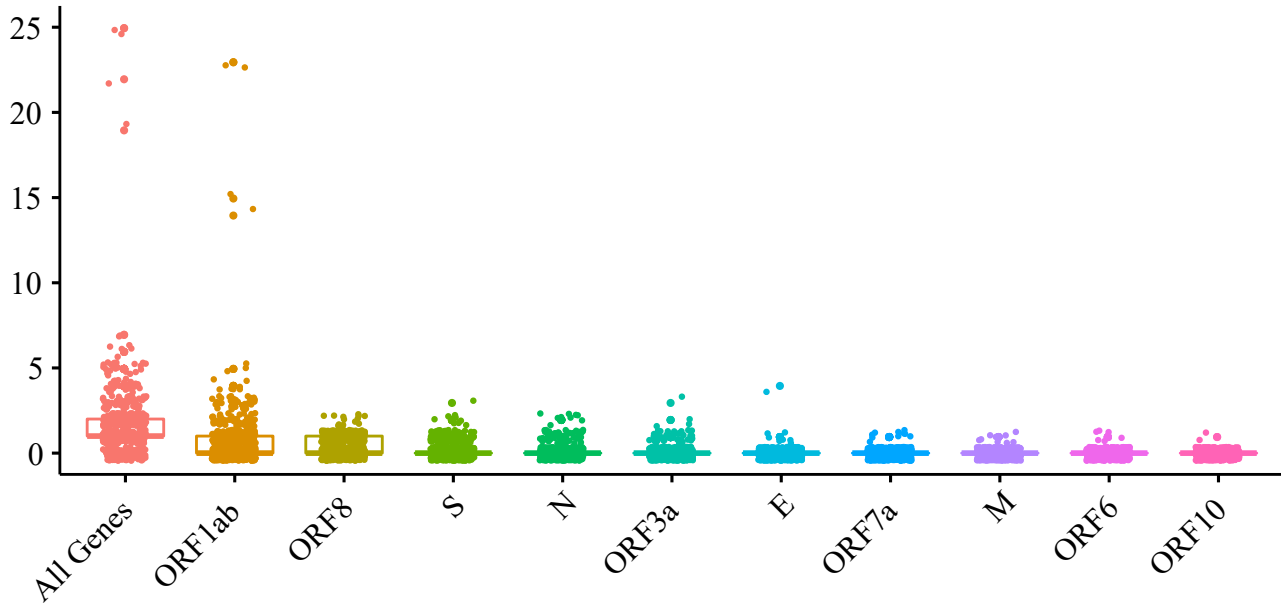

Supplement: Supplementary file 3 — Supporting information [file CTM2-11-e430-s006.pdf]

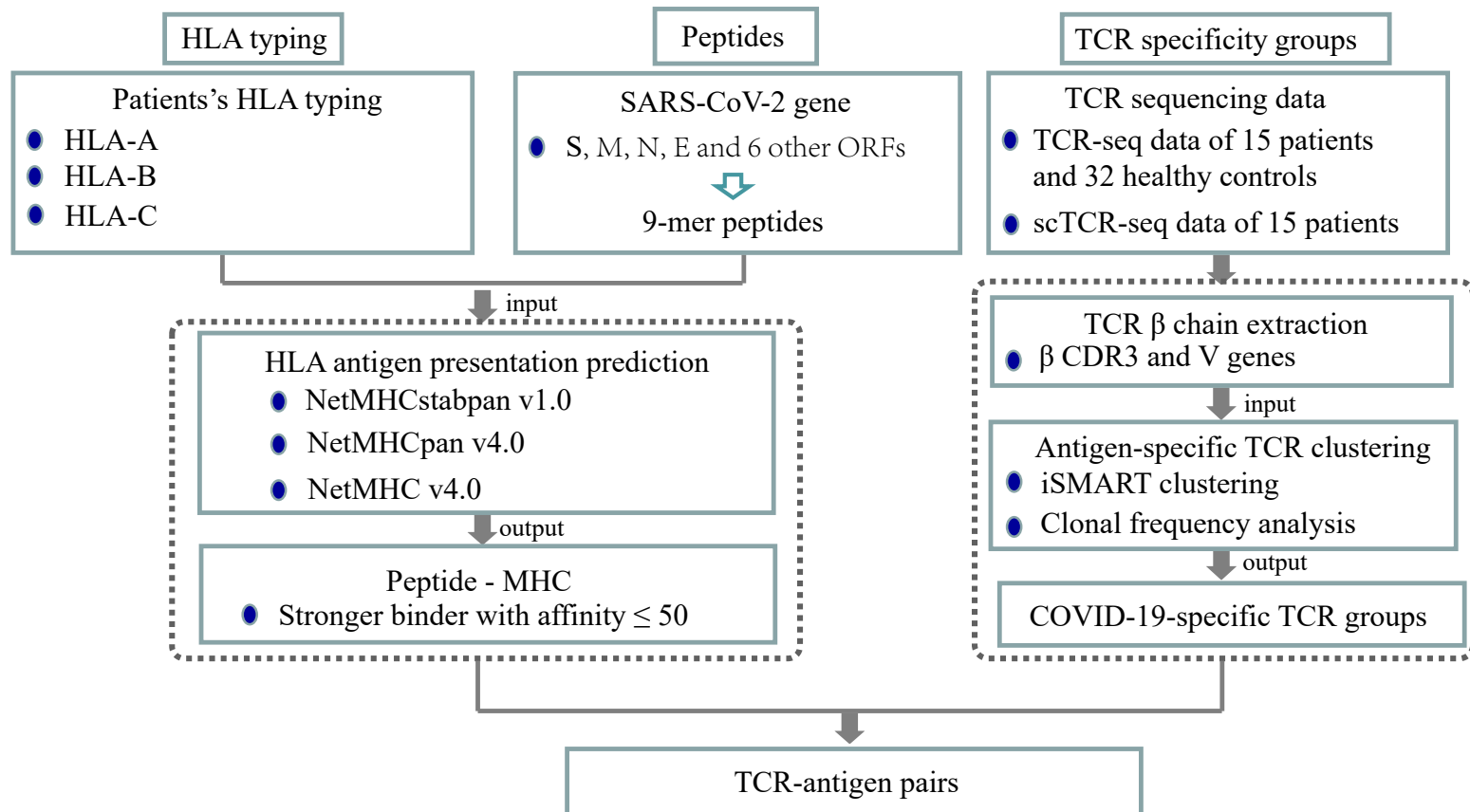

Supplement: Supplementary file 4 — Supporting information [file CTM2-11-e430-s004.pdf]
